# Experimentally Derived δ^13^C and δ^15^N Discrimination Factors for Gray Wolves and the Impact of Prior Information in Bayesian Mixing Models

**DOI:** 10.1371/journal.pone.0119940

**Published:** 2015-03-24

**Authors:** Jonathan J. Derbridge, Jerod A. Merkle, Melanie E. Bucci, Peggy Callahan, John L. Koprowski, Jean L. Polfus, Paul R. Krausman

**Affiliations:** 1 School of Natural Resources and the Environment, University of Arizona, Tucson, Arizona, United States of America; 2 Jerod A. Merkle, Département de Biologie and Centre d'Étude de la Forêt, Université Laval, Québec, Canada; 3 Boone and Crockett Program in Wildlife Conservation, Wildlife Biology Program, University of Montana, Missoula, Montana, United States of America; 4 Wildlife Science Center, Forest Lake, Minnesota, United States of America; 5 Natural Resources Institute, University of Manitoba, Winnipeg, Manitoba, Canada; Michigan Technological University, UNITED STATES

## Abstract

Stable isotope analysis of diet has become a common tool in conservation research. However, the multiple sources of uncertainty inherent in this analysis framework involve consequences that have not been thoroughly addressed. Uncertainty arises from the choice of trophic discrimination factors, and for Bayesian stable isotope mixing models (SIMMs), the specification of prior information; the combined effect of these aspects has not been explicitly tested. We used a captive feeding study of gray wolves (*Canis lupus*) to determine the first experimentally-derived trophic discrimination factors of C and N for this large carnivore of broad conservation interest. Using the estimated diet in our controlled system and data from a published study on wild wolves and their prey in Montana, USA, we then investigated the simultaneous effect of discrimination factors and prior information on diet reconstruction with Bayesian SIMMs. Discrimination factors for gray wolves and their prey were 1.97‰ for δ^13^C and 3.04‰ for δ^15^N. Specifying wolf discrimination factors, as opposed to the commonly used red fox (*Vulpes vulpes*) factors, made little practical difference to estimates of wolf diet, but prior information had a strong effect on bias, precision, and accuracy of posterior estimates. Without specifying prior information in our Bayesian SIMM, it was not possible to produce SIMM posteriors statistically similar to the estimated diet in our controlled study or the diet of wild wolves. Our study demonstrates the critical effect of prior information on estimates of animal diets using Bayesian SIMMs, and suggests species-specific trophic discrimination factors are of secondary importance. When using stable isotope analysis to inform conservation decisions researchers should understand the limits of their data. It may be difficult to obtain useful information from SIMMs if informative priors are omitted and species-specific discrimination factors are unavailable.

## Introduction

What do free-ranging animals eat? Clear answers to this basic ecological question can be elusive; yet an understanding of simple trophic interactions is essential to management and conservation. For example, identifying the diet of black bears (*Ursus americanus*) that forage within human-dominated landscapes dictates management actions employed to reduce human-bear conflicts [1]. A variety of methods can be used to assess feeding ecology (e.g., stomach analysis [2], scat analysis [3]), but stable isotope analysis (SIA) is increasingly popular because it can more comprehensively characterize diets over temporal and spatial scales [4]. Although inference from SIA has benefited from recent statistical refinements [5–7] key methodological foundations remain to be addressed [8, 9].

Stable isotope mixing models (SIMMs) are used to describe and test hypotheses about direct trophic interactions among species [10, 11]. Inherent in SIMMs, are multiple sources of error that influence estimates of the proportional contribution of different prey to a consumer’s diet [12, 13]. The primary error source is the natural variation in ratios of heavy to light isotopes of C (δ^13^C) and N (δ^15^N) among individuals within a species [12, 14] which arises from variation in diets [15]. Another well-documented error source, termed trophic discrimination, comes from changes in δ^13^C and δ^15^N (i.e., Δδ^13^C and Δδ^15^N) that occur as prey tissues are incorporated into consumer tissues [16]. For specific tissue types (e.g., hair, muscle, collagen), trophic discrimination factors (TDFs) are the difference between δ^13^C and δ^15^N of a consumer and its food [7, 8], as lighter isotopes (e.g., ^12^C, ^14^N) are depleted during incorporation. Another important source of error comes from the specification of prior information in Bayesian SIMMs. Bayesian SIMMs are increasingly popular because of their ability to simultaneously incorporate multiple sources of variation [5, 13] and users must specify prior knowledge of the consumer’s diet, even if the knowledge is non-informative (i.e., diet source contributions are a priori constrained to be equally likely).

Herein, we examine two potential sources of error in Bayesian SIMMs: discrimination factors and prior information. The use of correct discrimination factors, given the species, tissue, and ecosystem in question, are often cited as the most important issue in SIA [17–19]. Model output can be sensitive to the discrimination factors used [8, 20], suggesting that species-, tissue-, and ecosystem-specific discrimination factors are required for unbiased and precise parameter estimates [19]. Experiments to determine species-specific TDFs are therefore necessary [17, 20, 21], but few studies have provided the factors and even fewer have been conducted on captive wild animals fed wild diets.

In Bayesian inference, prior information and the data influence results, and the contribution of each is based on their relative precision [22, 23]. Although, in the ideal case, data should swamp the priors, small sample sizes and non-species-specific TDFs are common problems in SIA studies, and in certain cases prior information could improve model performance [24]. For example, relatively high variance in individual prey and consumer stable isotope values and non-species-specific discrimination factors may provide Bayesian SIMM estimates that are highly variable [9] and possibly inaccurate [8] without the specification of unbiased and precise prior information to guide parameter estimation [5]. Currently, specifying prior information in SIMMs is optional in frameworks such as SIAR (Stable Isotope Analysis in R [13]). Although the mathematical details have been outlined [7, 24, 25] and examples of specifying priors from relevant data sources exist in the literature (e.g., diet proportions from gut contents of rainbow trout (*Oncorynchus mykiss*) [5], or from regurgitations fed to nestling double-crested cormorants (*Phalacrocorax auritus*) [26]), the majority of published SIMMs do not specify prior information. To our knowledge, a quantitative evaluation of the impact of prior information on SIMMs is not available.

Our objective was to investigate the simultaneous effect of discrimination factors and prior information on diet reconstruction with Bayesian SIMMs using gray wolves (*Canis lupus*) as a model consumer. We used an estimation procedure (see Calculating captive wolf diet) to determine the proportions of three species fed to captive wolves in a controlled feeding trial (hereafter, estimated diet). We then determined discrimination factors for gray wolves (hereafter, wolf discrimination factors). A previous study published wolf discrimination factors from an approximately controlled diet where wild wolves consumed moose (*Alces alces*) almost exclusively [27]. However, these values have not been widely used, in part because they were estimated for moose-wolf bone collagen and this tissue type is not typically available for studies of extant wild populations. We used our wolf discrimination factors alternately with experimentally-derived red fox (*Vulpes vulpes*) factors (hereafter, fox discrimination factors [28]) commonly used in wolf SIA studies [9, 29], and a gradient of priors that ranged from non-informative to minimum informative (i.e., the least precise prior information that resulted in SIMM estimates not significantly different from estimated diet) to compare their relative effects in characterizing the estimated proportional contribution of each prey source to wolf diet. Using estimated diet for comparison, we quantified bias, precision, and accuracy of posterior distributions at these varying levels of discrimination factors and prior information. Finally, to illustrate the varying effects of prior information and species-specific discrimination factors in a field setting, we re-analyzed a sample of recently published SIA data collected on wolves and their prey in northwestern Montana, USA [9].

## Materials and Methods

### Sample collection

We monitored the diets of 10 adult captive gray wolves (five F and five M) from 7 June 2011 to 31 May 2012 at the Wildlife Science Center, Forest Lake, Minnesota, USA. During this period, we recorded the number of white-tailed deer (*Odocoileus virginianus*), beaver (*Castor canadensis*) and Canada goose (*Branta canadensis*) consumed by specific individual wolves. Dominant wolves were kept separate from sub-dominant animals during feeding to ensure individuals had access to equal amounts of each food item. For each food item, we collected 5g of muscle tissue, and for deer, we also collected ≥100 guard hairs. We collected ≥50 guard hairs each from the rump and shoulder of wolves during capture events in November-December 2011 and May 2012 (May samples only included hairs remaining from the previous year’s growth). We stored all samples in a conventional freezer at approximately −20°C. All animal handling procedures were conducted according to the guidelines of the American Society of Mammalogists for use of wild mammals in research [30], and were specifically approved by the Institutional Animal Care and Use Committee of the Wildlife Science Center, Forest Lake, Minnesota, USA.

### Sample preparation and stable isotope analysis

We followed the methods of Derbridge et al. [9] to clean and prepare hair samples for SIA. Because lipids can affect stable isotope measurements [16, 31, 32], we prepared two <1-mm, <2-mg sub-samples from each 5-g muscle sample. We extracted lipids from one of each pair of tissue samples by rinsing it in a 2:1 chloroform/methanol solution three times. All muscle samples were rinsed with distilled water and dried in a laboratory drying oven at 60°C for ≥48 hr.

We sent samples to the Environmental Isotope Laboratory (Department of Geosciences, University of Arizona) for analysis of C and N stable isotopes on a continuous-flow gas-ratio mass spectrometer (Finnigan Delta PlusXL) coupled to a Costech elemental analyzer. We express isotope values in delta notation (δ) as:

$$\delta Z=\left(\frac{R_{\text{sample}}}{R_{\text{stamdard}}}-1\right)\times 1000$$

where *Z* is ^13^C or ^15^N, and *R* is ^13^C/^12^C or ^15^N/^14^N. Standardization was based on acetanilide for elemental concentration, NBS-22 and USGS-24 for δ^13^C, and IAEA-N-1 and IAEA-N-2 for δ^15^N. Based on repeated internal standards, precision was better than ± 0.10 for δ^13^C and ± 0.2 for δ^15^N.

### Calculating captive wolf diet

We assumed that food eaten after 31 October 2011 did not contribute to hair growth during the study period [33, 34] and estimated the proportion of each food item eaten between June and October for each individual wolf separately using a simple bootstrapping approach [35]. Using counts of each food item, multiplied by a weight sampled randomly from a uniform distribution of reported minimum and maximum weights of each species (41–223, 5–35, and 3–6 kg for deer [36], beaver [37], and goose [38], respectively), we developed an index of the total kg consumed during the hair growth period. We used this this index to calculate the proportion of each food item in the diet for each individual wolf 1,000 times. We verified that 95% confidence intervals of proportion estimates for each individual overlapped and then combined food item histories of all animals. We then used the bootstrapping procedure (sampling 1,000 times) to develop an estimate of the proportion of each food item to wolf diet.

### Estimating discrimination factors

Generally, SIMMs are developed to estimate the proportion *p* of each food source *s* (from 1 to *k* different sources) in the diet of each consumer *X* (from 1 to *i* individual consumers). However, to estimate discrimination factors between a consumer and its prey, we adapted a hierarchical Bayesian model used to estimate diet proportions [5]. Instead of estimating *p*, we estimated the discrimination factors *c* for each isotope of interest (from 1 to *j* different isotopes). The form of the normally distributed model and its overall combined variance was as follows:

$$\begin{array}{l} X_{ij}=\sum_{k=1}^{k}p_{k}\left(s_{jk}+c_{jk}\right)+\varepsilon_{ij}\\ \text{var}\left(X_{ij}\right)=\sum_{k=1}^{k}p_{k}^{2}\left(\omega_{jk}^{2}\right)+\sigma_{j}^{2} \end{array}$$

where *X*_*ij*_ was the estimated isotope value *j* of consumer *i* based on *k* sources. The residual error *ε*_*ij*_ described additional inter-observation variance not described by the model [6]. Although there has been some discussion over the appropriateness of the residual error term [6], we included it because residual error is routinely monitored in generalized linear regression. Furthermore, we estimated discrimination factors without the residual error term and discrimination value estimates and their SEs were < 0.02 different. The model distributions were: *s*_*jk*_ ~ *Normal*(*μ*_*jk*_, *ω*_*jk*_^*2*^), *p*_*k*_ ~ *Dirichlet*(*α*_*k*_), *ε*_*ij*_ ~ *Normal*(0, *σ*_*j*_^*2*^), where source values *s*_*jk*_ were normally distributed with mean *μ*_*jk*_ and variance *ω*_*jk*_^*2*^. *Proportions p*_*k*_ *were based on a Dirichlet distribution [39] with k* values of *α* corresponding to the distribution of estimated proportions of each dietary source. The residual error *ε*_*ij*_ was based on a normal distribution with a mean of 0 and variance *σ*_*j*_^*2*^. Both *c*_*jk*_ and *σ*_*j*_^*2*^ were estimated by the model. For *p*_*k*_, we estimated a vector of *α* parameters of the Dirichlet distribution with maximum likelihood, using our bootstrapped proportion data, in the package dirmult in program R 3.0.1 [40]. We specified vague priors for *c*_*j*_, based on a uniform distribution between 0 and 10.

We used Markov chain Monte Carlo (MCMC) methods to estimate the parameters of the mixing model, which produces simulations of plausible values of *c*_*k*_ and *ε*_*j*_ consistent with the data. We ran three parallel MCMC chains with a burn-in of 50,000 iterations. We generated posterior samples using 15,000 iterations of the model and a thinning rate of 15. We chose the number of iterations by calculating the Gelman and Rubin convergence diagnostic [41] and increasing the number of iterations until the statistic was <1.1. Parameterization of the mixing model was conducted in R 3.0.1 [40] and JAGS [42] using the R package rjags [43].

### Effects of varying priors and discrimination factors

Using our estimated wolf discrimination factors and fox discrimination factors along with varying prior information, we used a Bayesian SIMM to estimate the proportional contributions of each prey (hereafter, SIMM posteriors) and compared them to estimated diet. To derive SIMM posteriors, we followed the framework of Jackson et al. [6], and estimated the *p* of each prey source. Fox discrimination factors were 2.6‰ (SD = 0.282) for δ^13^C and 3.4‰ (SD = 0.204) for δ^15^N [9, 28].

We used our captive wolf diet estimates as the data source for informative priors in the Bayesian SIMM. Although somewhat circular, this approach was essential to exploring the effect of varyingly informative priors, and because wolf diet was controlled, we could consider our estimates, and therefore our priors, to be relatively unbiased. By contrast, priors for field studies contain inherent bias because researchers must choose them according to a subjective belief that they resemble the data [44]. Our level of control allowed us to manipulate informativeness of prior information to demonstrate how it affects SIMM posteriors from field studies.

We examined a gradient of priors from non-informative to minimum informative. We employed a stepwise approach to identify the minimum informative prior of source proportions that resulted in SIMM posteriors that were not different from estimated diet. Although other variants are available [7], priors for proportions are often incorporated using the Dirichlet distribution [13, 45]. We adjusted priors (i.e., α parameters of the Dirichlet distribution) to approximate the precision of the distribution of source contributions from our estimated captive wolf diet. Our approach to estimate the minimum informative prior was as follows: 1) we increased the α value of the source that was the most consumed (i.e., in our case, deer) one integer at a time until; 2) the ratio between the most consumed source and the second most consumed source equaled the ratio between the first α value and 1 (in our case, a rounded α = 24 for deer and 1 for goose); 3) we increased both α values while maintaining the ratio (i.e., 24:1) until the ratio between the 1^st^, 2^nd^, and 3^rd^ source reflected the estimated diet; 4) we continued this process (i.e., re-parameterizing proportional contributions of each prey species following methods above) until the SIMM posteriors did not differ from the distribution of estimated diets.

We used MCMC to parameterize mixing models with each set of discrimination factors and the varying Dirichlet distributions of prior information. We ran three parallel MCMC chains and found that a burn-in of 50,000 iterations, along with posterior sampling of 15,000 with a thin rate of 15, was sufficient for convergence [41]. We tested whether the SIMM posteriors of each prey species were different from estimated wolf diet by generating 1,000 proportion values of each prey species fed to wolves from the Dirichlet distribution with the estimated α vector. These 1,000 proportions were subtracted from a random sample of 1,000 posterior estimates of each proportion of the MCMC. We concluded that SIMM posteriors were not different from estimated diet when the differences of all three prey species’ 95% confidence intervals overlapped zero.

To estimate the influence of different discrimination factors and the level of accuracy required to specify priors, we monitored bias, accuracy, and precision of SIMM posteriors for each model. Based on 1,000 randomly selected SIMM posteriors *p̂*_*kt*_ and 1,000 bootstrapped estimates *p*_*kt*_, we calculated bias of each proportional estimate as:

$$\text{bias}\left({\hat{p}}_{k}\right)=\left[\left(\sum_{t=1}^{n}{\hat{p}}_{kt}-p_{kt}\right)\times \frac{1}{n}\right]$$

We calculated the Monte Carlo variance (i.e., precision) for each *p̂*_*k*_ estimate, following:

$$\mathrm{var}\left({\hat{p}}_{k}\right)=\left[\sum_{t=1}^{n}{\left({\hat{p}}_{kt}-\frac{1}{n}\sum_{t=1}^{n}{\hat{p}}_{kt}\right)}^{2}\right]\times \frac{1}{n-1}$$

where smaller variance estimates represent increased precision. Finally, we calculated accuracy as the mean squared error (MSE), following MSE(*p̂*_*k*_) = var(*p̂*_*k*_) + (bias(*p̂*_*k*_))^2^, where smaller values of MSE represent higher accuracy.

### Empirical illustration

Derbridge et al. [9] recently reported that wolves in northwestern Montana consume varying proportions of six prey species: white-tailed deer, mule deer (*Odocoileus hemionus*), elk (*Cervus canadensis*), moose, beaver, and snowshoe hare (*Lepus americanus*). They used fox discrimination factors [28], and compared models estimated with non-informative priors to models estimated with informative priors derived from the biomass of available prey within the study area [9]. We re-analyzed a sample of their data to illustrate how altering prior information and species-specific discrimination factors affects inference on the proportional contribution of prey to wolf diet.

Although Derbridge et al. [9] reported high variation among packs, we estimated a population-level diet to simplify our illustration. We used a random sample of δ^13^C and δ^15^N values from a single wolf from each of the 12 packs. Following the methods described above, we calculated the proportional contribution of each of the six prey species to wolf diet based on varying prior information and discrimination factors derived from four models: 1) non-informative priors and fox discrimination factors, 2) non-informative priors and wolf discrimination factors, 3) informative priors based on Derbridge et al. [9] and fox discrimination factors, and 4) informative priors and wolf discrimination factors. To examine the impact of prior information and discrimination factors on diet inference, we compared models 1, 2, and 3 to model 4 (i.e., for illustrative purposes we assumed model 4 was as close to the true diet as possible). Models were compared by subtracting the posterior samples of each prey species of models 1, 2, and 3 from model 4 and examining whether the 95% CI of the difference overlapped zero. In addition, we compared posterior distributions between the models by calculating bias, precision, and accuracy following the methods described above.

## Results

We fed wolves 241 food items (106 deer, 14 beaver, 121 geese). We used a subsample of 20 muscle and 23 hair samples from deer, 13 muscle samples from beaver, and 15 muscle samples from goose to calculate the distribution of δ^13^C and δ^15^N of prey. The analytical precision of the stable isotope estimates was ≤ 0.1 and 0.17 (1 SD) for δ^13^C and δ^15^N, respectively. We did not detect a difference between hair and muscle tissue samples of deer for δ^13^C (Welch’s t-test, *t*_*40*.*5*_ = 0.247, *P* = 0.806) and δ^15^N (Welch’s t-test, *t*_*40*.*9*_ = 1.408, *P* = 0.167). Therefore, we used muscle tissue from deer for further analyses. We detected differences between untreated and lipid-extracted muscle tissue of geese for δ^13^C (Paired t-test, *t*_*14*_ = -2.839, *P* = 0.013), and deer for δ^15^N (Paired t-test, *t*_*19*_ = 3.001, *P* = 0.007), and used only lipid-extracted samples in our analysis.

We collected 37 hair samples (19 rump, 18 shoulder) from the 10 wolves between the two collection periods (i.e., 10 wolves in Nov-Dec, 9 wolves in May [Table 1]). We did not detect a difference between the two sampling periods of wolf hairs for δ^13^C (rump, *F*_*1*,*17*_ = 0.004, *P* = 0.951; shoulder, *F*_*1*,*16*_ = 0.045, *P* = 0.835) or δ^15^N (rump, *F*_*1*,*17*_ = 0.727, *P* = 0.406; shoulder, *F*_*1*,*16*_ = 1.442, *P* = 0.247). Additionally, we did not detect a difference between wolf hairs collected from the rump or shoulder for δ^13^C (Paired t-test, *t*_*17*_ = 1.505, *P* = 0.151) or δ^15^N (Paired t-test, *t*_*17*_ = -0.748, *P* = 0.465). Therefore, we used a single rump sample from each of the 10 wolves sampled in November and December in subsequent analysis.

**Table 1:** Mean and SD of δ^13^C and δ^15^N for tissue samples collected from a subsample of white-tailed deer, beaver, and goose fed to gray wolves (*n* = 10) during a captive feeding study of wolves in Forest Lake, Minnesota, USA, 2011–2012.

			δ^13^C		δ^15^C	
Species	Tissue	*n*	Mean	SD	Mean	SD
Deer	Hair	23	−21.92	3.03	4.69	1.13
Deer	Muscle	20	−22.12	2.35	4.24	0.99
Beaver	Muscle	13	−24.80	0.34	2.32	1.60
Goose	Muscle	15	−25.57	1.46	4.00	1.18
Wolf	Hair (rump)	19	−20.38	0.64	7.16	0.30
Wolf	Hair (shoulder)	18	−20.04	0.53	7.09	0.26

### Estimating discrimination factors

Using our bootstrapping procedure, we estimated wolf diet to be 0.941 deer (SD = 0.003, 95% CI = 0.934–0.947), 0.020 beaver (SD = 0.002, 95% CI = 0.015–0.025), and 0.039 goose (SD = 0.002, 95% CI = 0.035–0.042). Using maximum likelihood, we estimated the α vector of the Dirichlet distribution of the diet of wolves to be 582.1, 12.4, and 24.3 for deer, beaver, and goose, respectively. Using this Dirichlet distribution as the proportional contribution of each prey and the distributions of prey muscle values (Table 1), we estimated the discrimination factors between gray wolves and their prey to be 1.97‰ for δ^13^C and 3.04‰, for δ^15^N (Table 2).

**Table 2:** Posterior density distributions of parameter estimates of a Bayesian stable isotope mixing model formulated to estimate discrimination factors of δ^13^C and δ^15^N between a gray wolves and their prey.

			95% CI	
Variable	Mean	SD	Lower	Upper
Discrimination (δ^13^C)	1.97	0.70	0.66	3.37
Discrimination (δ^15^N)	3.04	0.31	2.44	3.66
Residual error (δ^13^C)	0.47	0.40	0.08	1.57
Residual error (δ^15^N)	0.26	0.18	0.07	0.74

### Effects of varying priors and discrimination factors

**Fig 1:**
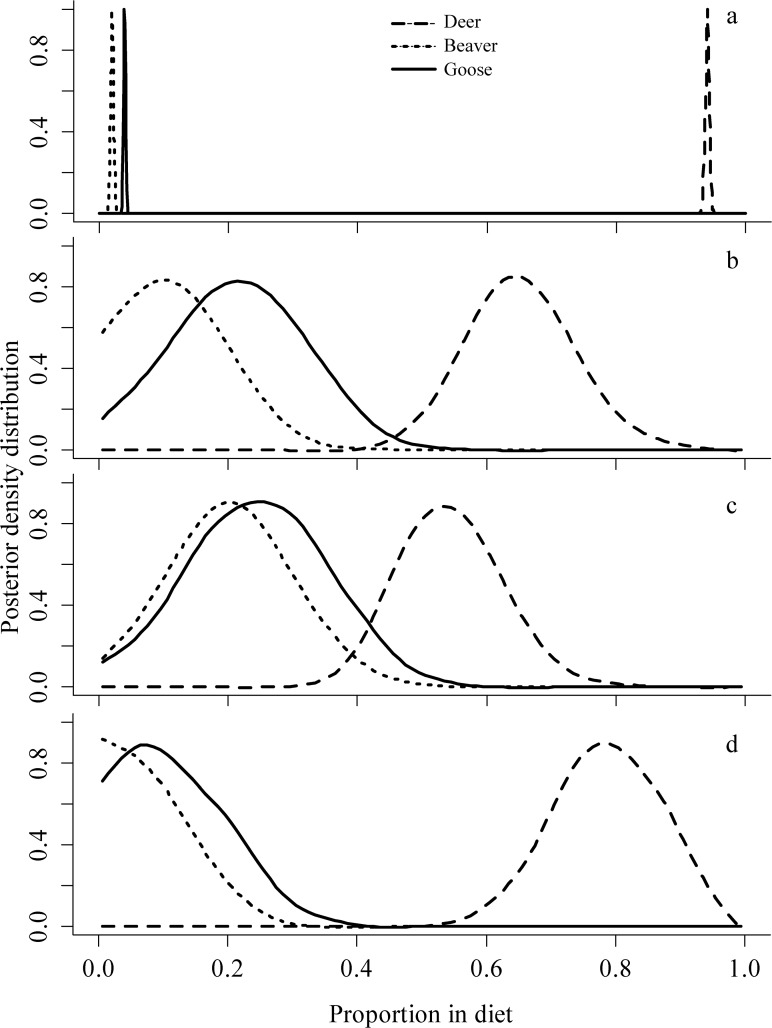
Distribution of proportional contributions of white-tailed deer, beaver, and Canada goose to the diet of gray wolves (*n* = 10) during a captive feeding study, Forest Lake, Minnesota, USA, 2011–2012. Diet (a) was estimated by bootstrapping the number of each prey (and the range of possible masses from the literature) fed to each wolf. Sections b, c, and d, represent posterior density distributions of Bayesian stable isotope mixing models formulated to estimate proportional contributions based on: non-informative priors (Dirichlet distribution α vector of 1,1, and1, for deer, beaver, and goose, respectively) and wolf discrimination factors estimated from this study (b); non-informative priors (same as b) and fox discrimination factors (c); and minimum informative priors (Dirichlet distribution with α vector of 13, 1, and 1) and wolf discrimination factors from this study (d).

Mixing models with varying priors and discrimination factors provided a variety of proportional contribution estimates (Table 3; Fig. 1). The model parameterized with fox discrimination factors and non-informative priors had diet proportions with the most bias and least precision. The difference between the mean estimated diet and the mean SIMM posterior distribution of proportions was approximately 0.4 lower for deer, 0.2 higher for beaver, and 0.2 higher for goose (Table 3). The model parameterized with wolf discrimination factors and non-informative priors decreased the MSE of proportion estimates by 46, 63, and 16% for deer, beaver, and goose, respectively; however, not all estimates were statistically similar to the estimated diet (e.g., mean proportions of deer were still > 0.28 different). Only after increasing the precision of the priors did all SIMM posteriors become similar to the estimated diet (i.e., the 95% CI of the difference between the SIMM posteriors and estimated values overlapped zero [Table 3; Fig. 1]).

**Table 3:** Estimates of the proportional contribution of white-tailed deer, beaver, and Canada goose to the diet of gray wolves (*n* = 10) during a captive feeding study in Forest Lake, Minnesota, USA, 2011–2012.

Type	Prey	Priors	Disc.	Sig.	Mean	SD	Bias	Variance	MSE
Estimated	Deer	NA	NA	NA	0.941	0.003	NA	NA	NA
SIMM	Deer	1,1,1	F		0.544	0.164	−0.397	0.007	0.164
SIMM	Deer	1,1,1	W		0.656	0.090	−0.285	0.008	0.089
SIMM	Deer	20, 1, 1	F	***	0.779	0.091	−0.162	0.008	0.034
SIMM	Deer	13,1,1	W	***	0.784	0.087	−0.158	0.007	0.032
Estimated	Beaver	NA	NA	NA	0.020	0.003	NA	NA	NA
SIMM	Beaver	1,1,1	F		0.215	0.094	0.195	0.009	0.047
SIMM	Beaver	1,1,1	W	***	0.124	0.078	0.104	0.006	0.017
SIMM	Beaver	20, 1, 1	F	***	0.117	0.074	0.098	0.005	0.015
SIMM	Beaver	13,1,1	W	***	0.091	0.065	0.072	0.004	0.009
Estimated	Goose	NA	NA	NA	0.039	0.002	NA	NA	NA
SIMM	Goose	1,1,1	F		0.242	0.104	0.203	0.011	0.052
SIMM	Goose	1,1,1	W	***	0.220	0.105	0.181	0.011	0.044
SIMM	Goose	20, 1, 1	F	***	0.104	0.076	0.064	0.006	0.010
SIMM	Goose	13,1,1	W	***	0.125	0.083	0.086	0.007	0.014

Proportional contributions to diet (Estimated; Mean and SD) were estimated by bootstrapping the number of each prey (and the range of possible masses from the literature) fed to each wolf. Proportional contributions (SIMM; Mean and SD) represent posterior density distributions of Bayesian stable isotope (δ^13^C and δ^15^N) mixing models formulated with various priors (from non-informative to minimum informative prior) and discrimination factors (i.e., red fox [F] and wolf [W]). In column Sig., *** indicates when the 95% CI of the difference between the estimated values and SIMM posteriors overlapped zero. Priors represent the vector of α values of a Dirichlet distribution corresponding to deer, beaver, and goose, respectively.

We estimated the minimum informative prior, using wolf discrimination factors, as an α vector of 13, 1, and 1 of the Dirichlet distribution (Table 3). The mixing model parameterized with these priors and discrimination factors provided proportion estimates with 80, 80, and 73% lower MSE than the mixing model parameterized with fox discrimination factors and non-informative priors. In other words, estimates of diet proportions were five times more accurate. In addition, the minimum informative prior necessary to estimate the correct (or at least statistically indistinguishable) diet using the fox discrimination factors corresponds to an α vector of 20, 1, and 1 (Table 3).

**Fig 2:**
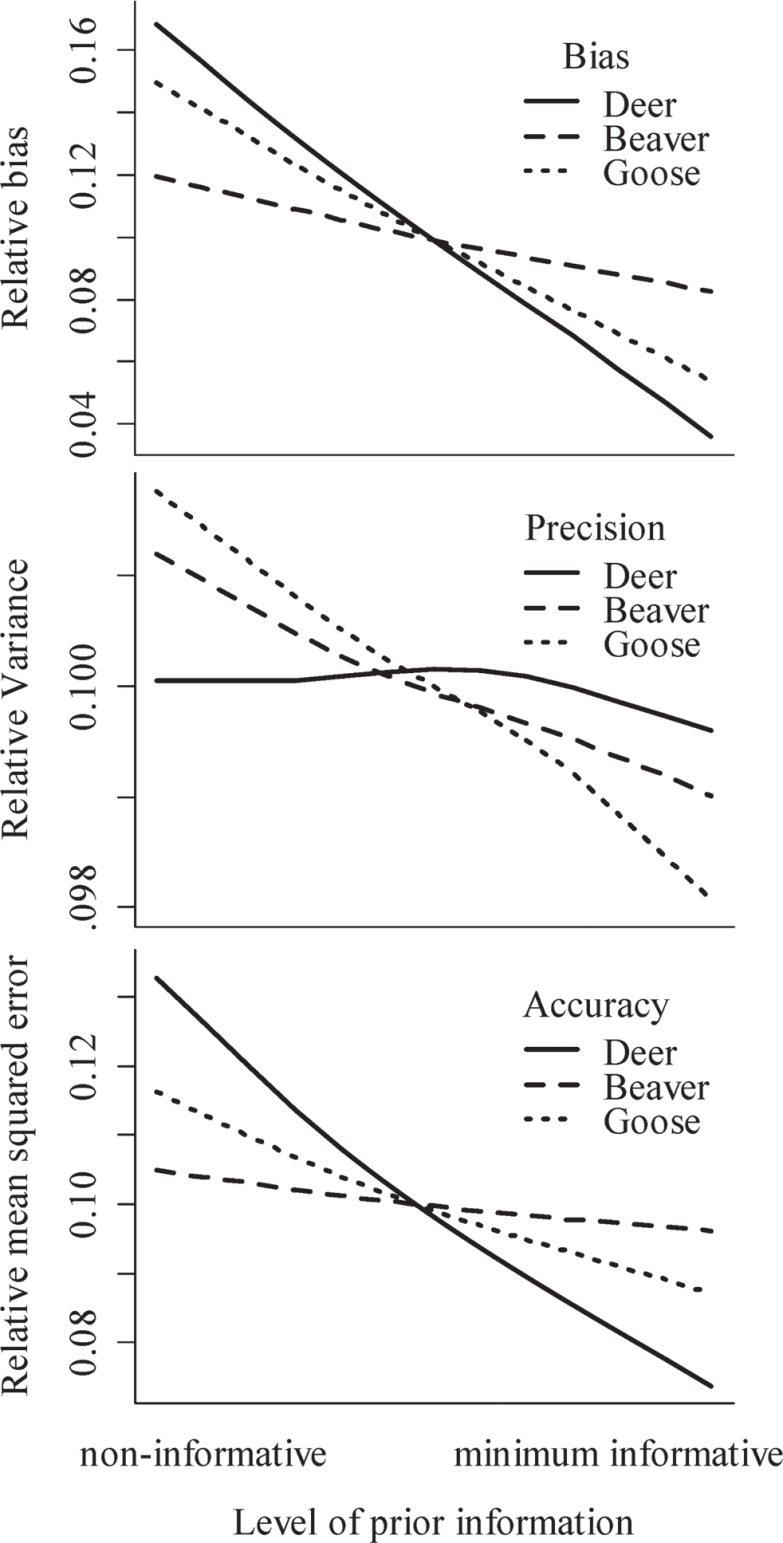
Estimates of the absolute value of bias, precision, and accuracy of parameter estimates from Bayesian stable isotope mixing models with varying levels of priors (i.e., from non-informative to the minimum informative prior). Parameters represent the proportional contributions of white-tailed deer, beaver, and Canada goose to the diet of gray wolves (*n* = 10) during a captive feeding study located at the Wildlife Science Center, Forest Lake, Minnesota, USA, 2011–2012. Mixing models were based on wolf discrimination factors estimated from this study. Non-informative priors correspond to a Dirichlet distribution with α vector of 1, 1, 1. Minimum informative priors represent the vaguest priors where SIMM posteriors were not statistically different from estimated proportions, with Dirichlet distribution α vector of 13, 1, 1.

The investigation of how bias, precision, and accuracy changed as prior information became more informative revealed inconsistent patterns. In general, increasing the precision of the priors decreased bias, and increased precision and accuracy (i.e., an inverse relationship with variance and MSE). However, for deer proportion estimates, increasing the precision of the priors had a stronger effect on bias than on precision (i.e., increasing the precision of the priors reduced variance in estimates for beaver and goose, but not deer). Overall, increasing the precision of priors had a greater effect on bias than precision (Fig. 2).

### Empirical illustration

For wolf diet in northwestern Montana, the proportional contribution of white-tailed deer, mule deer, elk, moose, beaver, and snowshoe hair varied depending on whether or not prior information was used and which discrimination factors were specified (Table 4). For example, model 1 (non-informative priors and fox discrimination factors) suggests that wolves consumed moose more than any other prey species, and that white-tailed deer was the least consumed species among the ungulate prey. However, model 4 (informative priors and wolf discrimination factors) suggests white-tailed deer comprised >50% of wolf diet, and moose was the least-consumed ungulate (Table 4).

**Table 4:** Mean (± SD) posterior distributions estimated from Bayesian stable isotope mixing models of the proportional contribution of six potential prey species to wolf diet in northwestern Montana, USA, 2009.

Priors	Disc.	W-t deer	Mule deer	Elk	Moose	Beaver	Hare
N	F	0.11 ± 0.08	0.17 ± 0.10	0.22 ± 0.14	0.33 ± 0.11	0.07 ± 0.05	0.10 ± 0.07
N	W	0.16 ± 0.13	0.24 ± 0.13	0.13 ± 0.12	0.28 ± 0.13	0.14 ± 0.08	0.05 ± 0.05
I	F	0.42 ± 0.07	0.10 ± 0.05	0.15 ± 0.06	0.15 ± 0.07	0.01 ± 0.01	0.17 ± 0.06
I	W	0.52 ± 0.07	0.11 ± 0.05	0.13 ± 0.06	0.10 ± 0.05	0.01 ± 0.01	0.13 ± 0.05

Four models were specified with two different prior distributions (i.e., non-informative [N], and informative [I]) and two different discrimination factors (i.e., red fox [F] and wolf [W]).

The comparison of proportional contributions to diet from models 1 (non-informative priors and fox discrimination) and 2 (non-informative priors and wolf discrimination) with model 4 revealed that specifying wolf discrimination factors, 1) decreased bias in three of the prey species but increased it in the others, 2) increased the variance in four of the species and decreased it in two, and 3) increased accuracy (MSE) in three of the six species (Table 5). Focusing on the proportional contribution of deer for example, specifying wolf instead of fox discrimination factors while using non-informative priors decreased bias by 12%, but increased variance by 400% (Table 5). Further, for both models with non-informative priors, the estimated posterior proportion of deer was significantly different from estimates of model 4 (our assumed true wolf diet).

**Table 5:** Comparison of posterior distributions estimated from Bayesian stable isotope mixing models of proportional contribution of six prey species to diet of gray wolves in Northwestern Montana, USA, 2009.

Prey	Priors	Disc.	95% CI of difference	Bias	Variance difference	MSE
W-t deer	N	F	0.19, 0.59	0.411	0.0025	0.1760
W-t deer	N	W	0.06, 0.58	0.362	0.0102	0.1453
W-t deer	I	F	−0.09, 0.29	0.102	0.0002	0.0149
Mule deer	N	F	−0.29, 0.15	−0.059	0.0076	0.0139
Mule deer	N	W	−0.41, 0.12	−0.135	0.0154	0.0365
Mule deer	I	F	−0.15, 0.15	0.002	0.0002	0.0031
Elk	N	F	−0.40, 0.16	−0.079	0.0166	0.0257
Elk	N	W	−0.34, 0.19	−0.004	0.0116	0.0145
Elk	I	F	−0.19, 0.14	−0.019	0.0010	0.0042
Moose	N	F	−0.49, −0.01	−0.243	0.0100	0.0718
Moose	N	W	−0.44, 0.08	−0.178	0.0136	0.0479
Moose	I	F	−0.23, 0.12	−0.052	0.0023	0.0077
Beaver	N	F	−0.19, 0.02	−0.066	0.0027	0.0072
Beaver	N	W	−0.29, 0.00	−0.128	0.0063	0.0230
Beaver	I	F	−0.03, 0.04	0.002	0.0001	0.0002
Hare	N	F	−0.14, 0.19	0.036	0.0016	0.0054
Hare	N	W	−0.07, 0.21	0.084	0.0003	0.0100
Hare	I	F	−0.18, 0.11	−0.035	0.0005	0.0043

Models were estimated with combinations of non-informative priors (N), informative priors (I), fox discrimination factors (F), and wolf discrimination factors (W). The three models represented in the table were compared to a fourth model (assumed to be closest to the true diet) specified with informative priors and gray wolf discrimination factors. Variance difference was calculated as the variance of each model subtracted by the variance of the fourth model.

Specifying informative priors had a much greater effect on estimates of the proportional contribution of diet. Using informative priors and fox discrimination factors (model 3) decreased bias, increased precision, and increased accuracy in the estimates of the proportional contribution of all six species. Focusing on the proportional contribution of deer for example, specifying informative priors while keeping fox discrimination factors constant, decreased bias by 75% and decreased variance by 92% (Table 5). Further, after specifying informative priors, the estimated posterior proportion of deer was not different from estimates of model 4 (our assumed true wolf diet).

## Discussion

The ability to determine what free-ranging animals consume has been greatly improved by the incorporation of uncertainty (e.g., TDFs and prior information) in Bayesian SIMMs, however species-specific TDFs are still widely unavailable, and informative prior information is rarely specified. Rather than focusing on quantifying diet of our captive animals, our primary objective was to investigate the potential effects of TDFs and priors on diet reconstruction with Bayesian SIMMs. We derived discrimination factors between wolves and their prey in a controlled feeding study where we knew stable isotope values of wolves and each prey item. However, after incorporating our derived discrimination factors into a typical Bayesian SIMM that included variation in stable isotope values of wolves and prey, we were unable to reproduce SIMM posteriors that were statistically similar to the estimated diet unless some amount of informative prior information was specified (Table 3). We found a similar pattern in our empirical illustration of wolf diet in Montana; specifying informative priors based on prey availability had a higher impact on bias, precision, and accuracy of estimates than specifying species-specific discrimination factors. These findings together suggest that the inclusion of prior information can have a substantial effect on estimated diet proportions of a consumer, and that future empirical studies using Bayesian SIMMs would benefit from the inclusion of appropriately developed priors (i.e., from a relevant separate data source).

The effect of including prior information in all cases was stronger than specifying species-specific discrimination factors. For example, parameterizing SIMMs using non-informative priors (in comparison to using our specification of the minimum informative prior) can result in a considerable increase in bias (up to a 109% increase), decrease in precision (up to a 67% decrease), and decrease in overall accuracy (up to a 211% decrease) of the posterior distribution of the proportional contribution of each prey species. Although species-specific discrimination factors appeared to have less impact on SIMM posteriors than prior information, our results do suggest that species-specific discrimination factors can improve bias and precision of estimates [8]. As wolf discrimination factors have been previously published from a field study that approximated a controlled feeding environment, researchers should decide which values are most appropriate for their study (e.g., where bone collagen of prey and wolves are available, studies may benefit from using values from Fox-Dobbs et al. [27]). However, our reported discrimination factors for gray wolves are the first experimentally-derived values available for the species, and should be considered for use in future SIA of diet for this commonly studied carnivore of broad conservation interest. Our study also used stable isotope data from hairs of wolves and their prey; SIA field studies of mammals typically use hairs [9, 29], thus our results will be more directly applicable to future work.

Using a similar approach to ours (i.e., employing the SIAR framework), Bond and Diamond [8] reported that changing discrimination factors resulted in significantly different estimates of seabird diet. However, their chosen discrimination factors, gleaned from the literature, had much larger ranges that those we used, and the source contributions to diet were very closely grouped in isotopic space (i.e., a challenging situation to resolve, but Yeakel et al. [25] provide a remedy). The smallest and largest differences in discrimination factors used were 1.92 and 2.92‰ for Δδ^13^C and 0.48 and 2.09‰ for Δδ^15^N [8]. We did not test such widely ranging differences in discrimination factors (i.e., the largest differences between the two sets of discrimination factors in our study were 0.9 and 0.4‰ for Δδ^13^C and Δδ^15^N, respectively), but our results suggest modest differences in estimates from red fox and wolf discrimination factors and, thus, highlight a more critical and less-studied source of uncertainty, that of prior information.

The striking impact of priors on parameter estimates from Bayesian SIMMs, as suggested by our data, should encourage greater attention to this component of analysis, in part because such information may be readily available and easily included as a model input. Although multiple approaches exist to produce prior information, we recommend a complete and thorough effort to establish reasonable priors. Researchers can obtain prior information by using data from the literature [22], preliminary investigation [23], prey availability [9, 25], a different dataset within the current study [5], or they can construct priors through data cloning [46]. For example, Moore and Semmens [5] used a bootstrap procedure on gut content of rainbow trout to estimate prior distributions of prey contributions to diet estimated from SIA of trout muscle tissues. Similarly, Doucette et al. [26] used regurgitated boluses fed to chicks of double-crested cormorants to estimate prior information assumed to predict adult diets. Inevitably, this subjective choice of priors implies a researcher’s belief in how closely the chosen prior information resembles the data [44]. A way to scrutinize this choice is to compare prior and posterior distributions after fitting models with non-informative and informative priors. If posterior distributions more closely resemble non-informative priors, the selected informative prior distribution may be problematic [47, 48]. For SIA of diet, no further progress can be made if species-specific discrimination factors are also unknown, as our reported compounding effect of weak priors and non-species-specific discrimination factors suggests.

Over the last few decades, much methodological progress has been made in stable isotope ecology [12], ultimately resulting in the application of Bayesian inference to SIMMs [5, 13]. Our work contributes to a growing literature that investigates model attributes and their impacts on inference from Bayesian SIMMs. We demonstrate that species-specific discrimination factors may only slightly improve inference unless a certain level of informative prior information is also specified. Applied ecological research on free-ranging animals must contend with numerous uncertainties, but future research using SIA to estimate diets will benefit from a careful and well-informed specification of prior information. Researchers who seek to inform conservation decisions with SIA should understand the limits of their data; when precise information is a central objective, SIA could be a futile exercise if informative priors are not incorporated and species-specific discrimination factors are unavailable.

## Supporting Information

S1 Table δ^13^C and δ^15^N of hair from 10 gray wolves, and of tissue samples from white-tailed deer, beaver, and goose they consumed during a captive feeding study, Forest Lake, Minnesota, USA, 2011–2012. (XLSX)
